# Supplementary material for: The causal relationship between the human gut microbiota and pyogenic arthritis: a Mendelian randomization study
Source: Front Cell Infect Microbiol. 2024 Nov 26;14:1452480. doi: 10.3389/fcimb.2024.1452480 (PMC11629706; doi:10.3389/fcimb.2024.1452480)
Supplement: Supplementary file 1 [file DataSheet1.zip › mendelian test/ebi-a-GCST90027723.csv_leave_one_out.pdf]

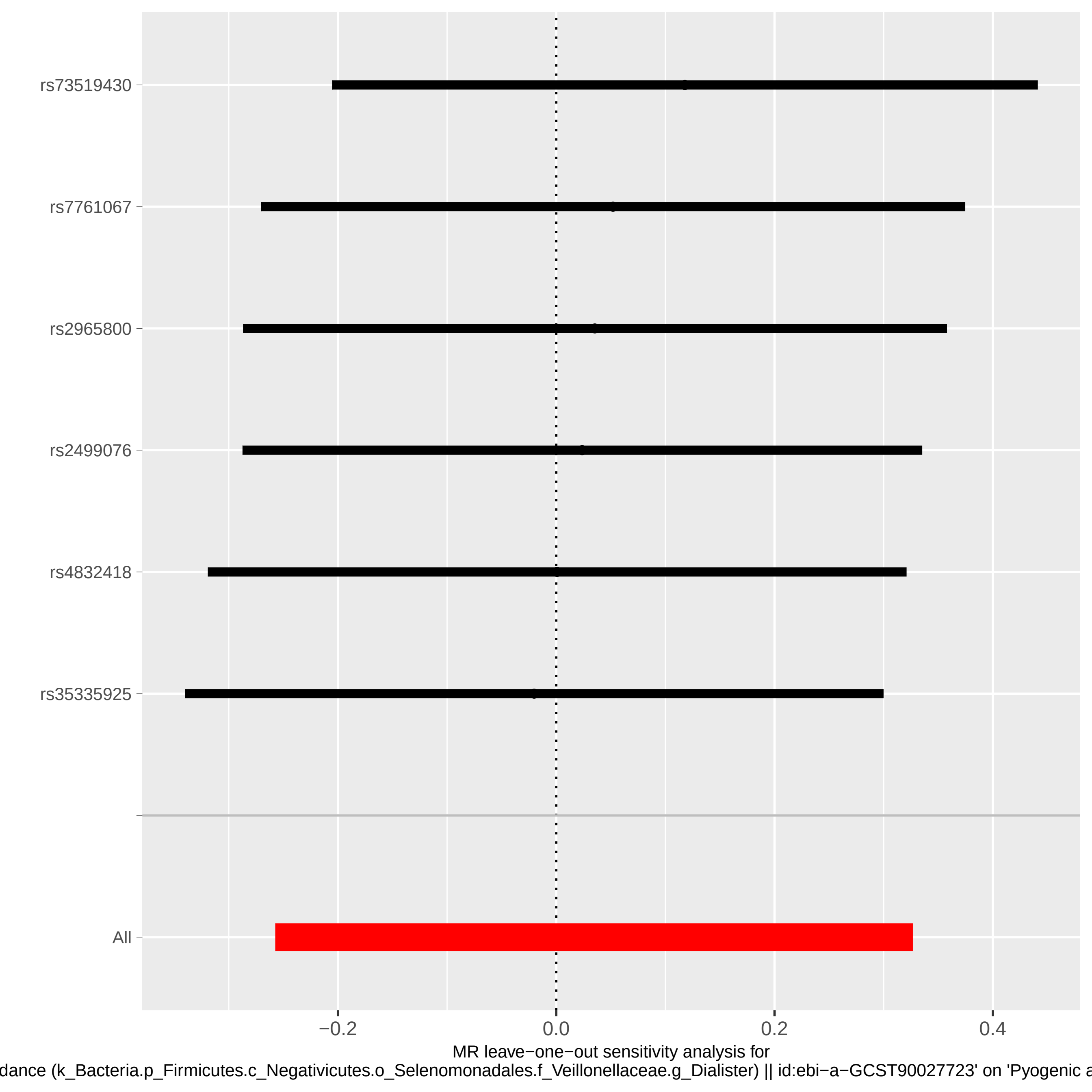
rs73519430
rs7761067
rs2965800
rs2499076
rs4832418
rs35335925
All
−0.2
0.0
0.2
0.4
MR leave−one−out sensitivity analysis for
dance (k_Bacteria.p_Firmicutes.c_Negativicutes.o_Selenomonadales.f_Veillonellaceae.g_Dialister) || id:ebi−a−GCST90027723' on 'Pyogenic a
